# Errata: Censo Brasileiro de Diálise 2023

**DOI:** 10.1590/2175-8239-JBN-2024-0081erpt

**Published:** 2025-04-25

No artigo “Censo Brasileiro de Diálise 2023”, com número de DOI: https://doi.org/10.1590/2175-8239-JBN-2024-0081pt, publicado no periódico Brazilian Journal of Nephrology, 47(1):e20240081, 2025, na página 6:

**Onde se lia:**

**Figura 6:**
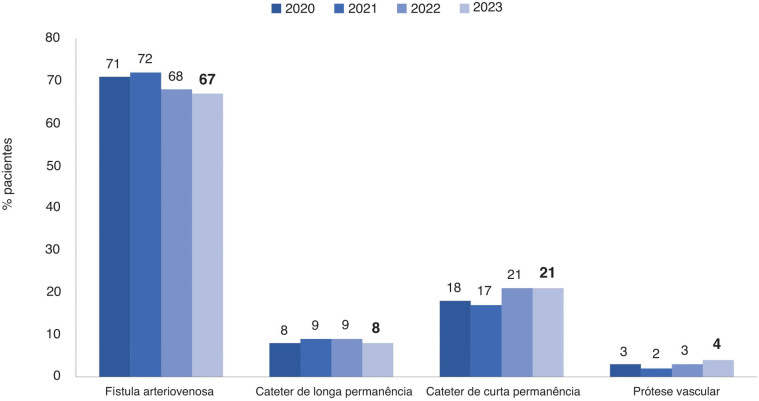
Tipo de acesso vascular usado para hemodiálise.

**Leia-se:**

**Figura 6:**
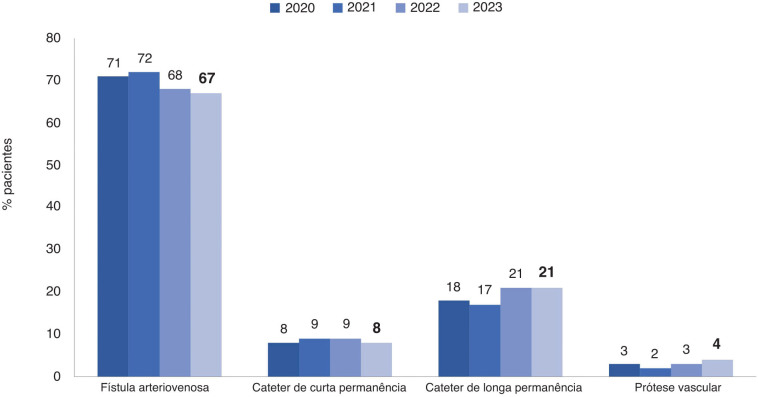
Tipo de acesso vascular usado para hemodiálise.

